# New Process for Tar-Water

**Published:** 1873-12

**Authors:** 


New Process for Tar Water.—L. Pommier prepares a concentrated tar water by macerating in a covered vessel for eight days a mixture consisting of ten parts each of Norwegian tar and ammonia water, and of one hundred parts of water; the mixture is then boiled to expel the excess of ammonia, then cooled and filtered. Thus prepared, it has a mild alkaline reaction to litmus, and may be diluted as required.

